# Influence of Housing and Management on Claw Health in Swiss Dairy Goats

**DOI:** 10.3390/ani11071873

**Published:** 2021-06-23

**Authors:** Lisa Marie Sailer, Mirjam Holinger, Joan-Bryce Burla, Beat Wechsler, Patrik Zanolari, Katharina Friedli

**Affiliations:** 1 Centre for Proper Housing of Ruminants and Pigs, Federal Food Safety and Veterinary Office FSVO, Agroscope Tänikon, 8356 Ettenhausen, Switzerland; joan-bryce.burla@agroscope.admin.ch (J.-B.B.); beat.wechsler@agroscope.admin.ch (B.W.); katharina.friedli@greenmail.ch (K.F.); 2 Department of Livestock Sciences, Research Institute of Organic Agriculture FiBL, Ackerstrasse 113, 5070 Frick, Switzerland; mirjam.holinger@fibl.org; 3 Clinic for Ruminants, Vetsuisse-Faculty, University of Bern, Bremgartenstrasse 109a, 3012 Bern, Switzerland; patrik.zanolari@vetsuisse.unibe.ch

**Keywords:** claw lesion, dairy goat, housing, locomotor activity, management, overgrown wall horn

## Abstract

**Simple Summary:**

Swiss dairy goats are commonly housed on deep littler, at least during the winter season. Due to the lack of horn wear, their claws often show overgrown wall horn. However, little is known about the impact of wall horn overgrowth on claw health and whether wall horn overgrowth impairs the goats’ locomotion behavior. Data was collected on 28 Swiss commercial dairy goat farms during autumn and spring. It was shown that severe wall horn overgrowth dominated during the indoor housing period and was associated with an increase in the proportion of claws with sole hemorrhages. Furthermore, wall horn overgrowth did not seem to have an effect on the goats’ locomotion behavior. The results of this study underline the importance of regular and careful claw trimming.

**Abstract:**

Due to a rising demand for goat milk and goat milk products worldwide, it is likely that dairy goat production will be intensified in the future, with larger herds per farm. In Switzerland, as in many other countries with intensive farming systems, dairy goats are typically housed on deep litter, with little access to hard abrasive surfaces. Such housing conditions will result in wall horn overgrowth. The aim of this study was to gain profound knowledge on the occurrence of overgrown wall horn, its impact on claw health and locomotor behavior, and possible adverse effects on animal welfare. Additionally, housing and management factors that may contribute to non-physiological claw conditions were evaluated. To compare claw conditions after the summer grazing period and the winter indoor housing period, data were collected on 28 Swiss dairy goat farms in autumn and spring (621 goats in total). Claw lesions were recorded with the help of a “claw card” documenting each claw. Furthermore, pictures were taken of each claw to determine the severity of wall horn overgrowth. Locomotion behavior (activity, lying time and lying bouts) was recorded with three-dimensional accelerometers fixed to the goats’ hind legs. In autumn, 66.7% of the examined claws showed moderate overgrowth, 32.4% severe overgrowth and 0.9% no overgrowth. In spring, 47.4% of the examined claws were affected with moderate overgrowth, 52.6% with severe overgrowth and 0.0% with no overgrowth. Horn separation (48.1% of examined claws) and sole hemorrhages (16.0% of examined claws) were the most frequent lesions. In goats with severely overgrown claws, the risk of developing sole hemorrhages was doubled compared with moderate overgrowth. The occurrence rate of horn separation was lower if the trimmer had attended a special skills training course (*p* < 0.001). Furthermore, locomotor activity (*p* < 0.01) and the number of lying bouts per day (*p* < 0.01) were higher in spring than autumn. Neither the goats’ activity nor the number of lying bouts per day differed before and after claw trimming. Finally, season and trimming were not associated with the goats’ total lying time. A certain extent of wall horn overgrowth in dairy goat claws cannot be avoided under the housing conditions typical for Swiss farms. Severe wall horn overgrowth is associated with an increase in the proportion of claws with sole hemorrhages. Therefore, regular and careful functional claw trimming, taking the housing situation (deep bedding, access to pasture, grazing on alpine pasture) into account, should be promoted.

## 1. Background

Goat milk production is on the rise worldwide due to increasing consumer demand and strong prices [1]. Goat milk is an alternative for people with cow’s milk intolerance (i.e., lactose intolerance) [2], and goat milk products are also becoming increasingly popular with gourmets. In 2000, the Bulletin of the International Dairy Federation recorded 14.2 billion tons of produced goat milk worldwide; in 2017, this figure had risen to 18.9 billion tons [3]. From 2000 to 2018, the global goat herd had increased by more than 37% to more than one billion goats [4]. In Canada, for example, the number of goats rose by 2.0% from 2011 to 2016. During this period, the number of farms declaring goats in Canada also fell by 5.4%, indicating that herd size per farm had increased [5]. The same phenomena can be seen e.g., in Greece, where fewer but larger farms were counted in 2000 until 2013 [1]. In accordance with this trend, the number of dairy goats rose by 3% from 2016 to 2018 in Switzerland, and the dairy goats’ performance level has increased on Swiss farms over the last 18 years [6,7].

As far as production systems for dairy goats are concerned, two main categories can be distinguished. Extensive systems relying mainly on grazing predominate in countries like Bangladesh, India and Sudan where the goats are farmed by smallholders for subsistence [8]. In contrast, intensive (indoor) farming is more common in most European and North American countries [9]. Europe has only 2.5% of the world’s goat herds but produces 18% of the world’s goat milk [5]. Spain has increased its goat milk production by 68,900 thousand metric tons in the years 1999 until 2009 and France has even risen its goat milk production by 127,660 thousand metric tons in the same time span [10].

A high prevalence of wall horn overgrowth and lameness has been reported in indoor systems [11,12,13]. Goat claws have evolved in semiarid regions, i.e., in an environment where even wear of the claw horn is guaranteed [9]. In contrast, goats kept indoors on deep bedding have a high prevalence of overgrown wall horn due to a lack of claw horn wear on hard abrasive material, even if the claws are trimmed several times a year [11,14]. In Switzerland, as in many other countries with intensive goat farming (New Zealand, France, Canada), dairy goats are typically housed on deep bedding without continuous access to a hard soil surface [15,16,17].

Overgrown wall horn can result in claw deformation and claw lesions [18]. Moreover, overgrown wall horn causes abnormal gait as well as undue stress on joints, tendons and ligaments and, in consequence, can lead to lameness [19], which also impairs animal welfare and reduces milk yield [12]. Finally, overgrown wall horn possibly increases the risk of infectious claw diseases, because manure might adhere underneath the protruding wall horn, thus providing optimal conditions for the propagation of various pathogens. In the scientific literature, there are few reports on *Dichelobacter nodosus* (foot rot) and *Treponema* spp. infections (Mortellaro) in goats in the United Kingdom [20,21], Brazil [22] and New Zealand [23,24]. However, little is known about infectious claw diseases in goats in general and their prevalence in Switzerland.

For dairy cows, detailed information on ideal claw conformation (the physical dimensions and the shape of the hoof) is available and has led to trimming schemes that aim at an even weight distribution between the medial and lateral claws of the hoof [25,26]. Studies have also shown that dairy cows with poor hoof conformation are predisposed to hoof lesions and lameness [27,28]. However, information is scarce about the specific morphology of goat claws. Claw measurements seem to vary between meat and dairy goat breeds, and front claws were found to be larger than hind claws [29]. Grenho Gonçalves Ajuda et al. [30] reported that 58% of 152 assessed goat feet were deformed and showed a positive correlation between a lameness score and the number of deformed claws.

With goat milk production trending upwards [1], an increase in the number of goat farms, herd size per farm, and milk yield level is to be expected in the future in Switzerland. Claw problems, including infectious claw diseases, could thus take on greater significance. Moreover, little is known about the quality of claw trimming in goats on Swiss farms, because claw trimming in goats is less organized and standardized than in dairy cows.

The aim of this study was to investigate claw health and claw characteristics in dairy goats kept in loose housing systems, based on data collected on 28 Swiss farms. Differences regarding the appearance and severity of wall horn overgrowth, the upcoming of claw lesions and changes in locomotion behavior were expected between the indoor housing (spring) and pasture grazing (autumn) period. During the indoor housing period, more and more severe wall horn overgrowth, due to lack of claw horn wear on abrasive surfaces was expected. Additionally, as the result of severe wall horn overgrowth, more claw lesions were expected. Furthermore, less locomotor activity, less lying bouts and more lying time were expected during the indoor housing period due to discomfort and pain caused by the before mentioned wall horn overgrowth and claw lesions. On each farm, claw condition was assessed in 12 goats, based on claw measurements as well as recordings of the amount of overgrown wall horn and the presence of slipper foot. Additionally, the occurrence of claw lesions and infectious claw diseases (*D. nodosus*, *Treponema* spp.) was determined. Finally, to compare the goats’ locomotion behavior before and after claw trimming, the 12 focal goats were equipped with accelerometers. In the analysis, the effects of overgrown wall horn on the occurrence of claw lesions and the goats’ locomotion behavior were evaluated. Moreover, housing and management factors were related to the occurrence of non-physiological claw conditions and impaired claw health.

## 2. Methods

### 2.1. Farms and Animals

The study was conducted on 28 Swiss dairy goat farms. The farms were recruited through public advertisement (homepage of the Swiss Goat Breeders Association, newsletters of the Swiss Consulting and Health Service for Small Ruminants and the Forum for Small Ruminants) or personal requests. Farms qualified for the survey if they had a loose housing system and a herd of more than 12 goats. The herd size on the participating farms ranged from 20 to 260 goats.

Before visiting each farm, 12 goats were randomly selected to be included in the study, using a modified version of the method proposed by Quirk [31]. For this purpose, the farmers provided a list with all animals that had been in lactation at least once. Randomly generated numbers were assigned to these animals. After these numbers had been sorted in an ascending order, the goats with the first 12 numbers were selected as focal animals. If there was more than one breed on the farm, the sample of goats selected as focal animals per breed was balanced as much as possible. During the first visit on the farm, the 12 preselected goats were inspected by a veterinarian (Lisa Sailer) to detect any apparent disease (i.e., mastitis, diarrhea, bad general health status). If one of these goats had a health problem, it was replaced by another goat. To reflect the variety of breeds on commercial dairy goat farms in Switzerland, all common breeds were represented in this study, including Chamois Colored (174 goats), Saanen (129), cross breeds (84), Toggenburger (65), Appenzeller (50), Grisons Striped (18), Peacock (16), Valais Blackneck (13), Capra Grigia (9), Nera Verzasca (8) and Anglo-Nubian (1). There were herds with either only horned (*n* = 3) or only hornless (*n* = 12) goats as well as mixed herds (*n* = 13).

The data for housing and claw trimming management were collected with a questionnaire (see File S1, Table S1). The housing of the animals varied from farm to farm. Twenty-five farms (89.3%) housed their animals on deep litter (straw) and three farms (10.7%) offered different bedding. Additional hard surfaces inside the barn were available on 27 farms (96.4%), and on 26 farms (92.8%) the goats had access to an outdoor exercise yard. Goats had access to pasture in summer on 27 farms (96.4%). On 11 farms (39.3%), goats had a free-range period on alpine pasture during summer. Farm management (e.g., housing, handling, feeding times and regimen, milking times) remained the same during the study.

The number of routine claw trimmings per year ranged from one to six, with an average of 3.1 times per year. The age of goats on occasion of the first trimming ranged from 1 to 20 months with a mean of 7.1 months. On most farms, the claws of the 12 focal goats were trimmed by the farmer himself. Twenty-four farmers (85.7%) in autumn and 22 farmers (78.6%) in spring trimmed the goats on their own. Two farm employees (7.1%) trimmed the claws in autumn and four (14.3%) in spring. Only on two farms (7.1%) and in autumn, the farmer engaged an external trained person to conduct the trimming. Eight trimmers (28.6%) in autumn and six trimmers (21.4%) in spring had attended special skills training for small ruminants. Equipment used for trimming were foot shears, pruning shears, hoof knives, pocket knives and angle grinders. Footbaths were used on none of the farms. None of the trimmers disinfected the trimming tools between animals. Bleeding lesions caused by trimming were disinfected on seven farms in autumn and on two farms in spring. On none of the farms in this study were bandages used to cover the bleeding. All results for housing and management per farm are presented in File S2.

### 2.2. Data Collection

Data collection took place between September 2018 and July 2019. Each farm was visited on a day for which the farmer had scheduled routine claw trimming in autumn and in the following spring. In autumn, after the grazing period, 336 goats were trimmed, and in spring, after the indoor housing period, 272 goats were trimmed. The loss of goats between autumn and spring was due to two farms not being included in the spring data collection (24 goats; one farm due to family-related reasons; on the second farm, the claws had been trimmed 1 week before the scheduled trimming date), slaughter (19), death (6), euthanasia (4) and sale to other farms (11).

### 2.3. Claw Conditions

During the trimming, claw conditions and claw lesions were recorded in detail, and the quality of the trimming was evaluated. To describe claw conditions, several measurements of the claws were made, and the amount of overgrown wall horn and the presence of slipper foot were recorded.

The medial and lateral digit of the front left and hind right leg were measured before and after the trimming by means of a measuring tape, using a modified version of the method proposed by Bhardwaj et al. [32].

**Figure 1:**
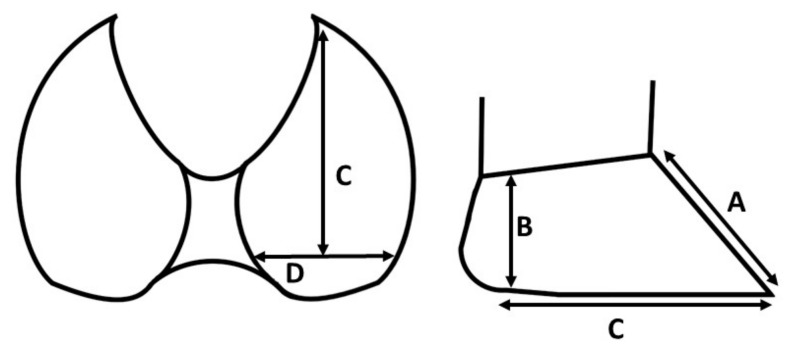
Schematic representation of the claws from plantar and lateral with the measurements: A = toe length, B = heel height, C = sole length, D = sole width.

The following parameters were recorded (Figure 1):

A. Toe length: Distance between the dorsal coronary band and the apex of the digit.
B. Heel height: Vertical distance from the sole to the coronary band at the extreme plantar or palmar margin of the heel on the floor.
C. Sole length: Length of the abaxial wall and bulb that are in contact with the floor surface.
D. Sole width (lateral and medial digit): Largest distance between the abaxial and axial wall at the sole–heel junction of each digit.

To determine wall horn overgrowth and slipper foot, three pictures of each foot (from lateral, from dorsal and from plantar) were taken before and after the trimming, with a Canon Power Shot 12G (Zoom Lens 5 × 15). Wall horn overgrowth was scored by means of the pictures taken from the plantar aspect. Overgrowth was classified by the sole surface covered by overgrown wall horn, as described in Table 1. The scores were assigned visually. In ambiguous cases, the amount of overgrown wall horn was calculated by means of a computer program (ImageJ 1.5, developed by NIH and LOCI, University of Wisconsin, Madison, WI, USA). Additionally, each animal was assigned an overall score of wall horn overgrowth (by adding up all eight severity scores). Thus, the lowest possible overall score of wall horn overgrowth was 0 (no overgrowth on any of the claws of one animal), and the highest 16 (all claws showing severity of wall horn overgrowth score 2). Finally, the direction and the origin of wall horn overgrowth were documented according to Table 1.

Regarding the presence of slipper foot, a foot was recorded as affected if one or both claws showed chronic overgrowth. This non-physiological claw form is also known as ingrown claw and defined as a chronic overgrowth of horn resulting in a long curling toe [11].

### 2.4. Claw Lesions

Claw lesions were recorded during the trimming and by means of a modified version of the claw card proposed by Lottner [33]. On all farms, the same veterinarian filled out the claw cards. Only trimmed claws were considered, because non-trimmed claws could not be inspected properly for all lesions. For each goat, a separate claw card was filled out. A claw card (translated to English) can be seen in File S3. On the claw card, all four feet were visualized and divided into the medial and lateral claw. Underneath each claw, the lesions were listed to tick if present. Furthermore, for all lesions, a subdivision into before (prior) and after (post) trimming was made. The following claw lesions were listed on the claw card: bleeding due to trimming, chronic laminitis, foreign body, granulomatous lesion, heel horn erosion, horn fissure, horn separation with an extra note if the farmer excised the loose wall horn completely, interdigital phlegmon, sole hemorrhage, sole/toe abscess, and sole/toe ulcer. In the case of horn separation, excising the loose wall horn completely is according to claw trimming guidelines [34].

Additionally, the condition of the interdigital space was recorded on the claw card using score 0 (unobtrusive), score 1 (mild dermatitis without necrosis) or score 2 (severe dermatitis with necrosis). Again, the same veterinarian who filled out the claw cards evaluated the condition. A detailed definition of the lesions is given in Table 2. All findings noted on the claw cards were verified using the pictures taken during the trimming, and doubtful findings were discussed with another specialist (K.F., P.Z.).

If there was suspicion of an infectious claw disease, particularly *D. nodosus* or *Treponema* spp. *(T. phagedenis*, *T. medium* and *T. pedis)*, cotton swab samples were taken of this particular interdigital space lesion, as described by Stäuble et al. [39]. The swabs were placed in tubes with buffer solution and sent to the Central Diagnostic Laboratory, Department of Clinical Veterinary Medicine, Vetsuisse-Faculty, University of Bern, for competitive real-time polymerase chain reaction analysis.

### 2.5. Lying Behavior and Locomotor Activity

Before and two weeks after trimming, the 12 focal goats on a given farm were equipped with an MSR data logger. To fix the logger to the leg, it was mounted to a 2 cm wide Velcro strap. To prevent pressure sores and compression of the blood vessel, a cotton pad was placed between the goat’s skin and the strap with the logger. Additionally, a self-adhesive bandage was wrapped on top to avoid loss and damage of the logger and to protect it against moisture and dirt. The loggers were set to one measurement per second and recorded the goats’ activity over 72 h. All goats had at least 12 h of habituation time before recording. No logger was removed by a goat.

Lying behavior and locomotor activity (standing and moving) were measured automatically by three-dimensional accelerometers (MSR145 data logger; MSR Electronics GmbH, Seuzach, Switzerland) fixed parallel (y-axis of the logger) to the metatarsus of the goat’s right hind leg. These accelerometers have been validated and used for the recording of locomotor activity in goats in previous studies [40,41]. The MSR data loggers recorded acceleration in *g* (m/s^2^) in vertical, horizontal and lateral direction of the leg. Thereby, the position of the leg in space, as well as changes in acceleration, are determined continuously. Changes in activity, e.g., from walking standing and lying could be calculated with the acceleration values. Values of 0 reflected a horizontal position of the logger and values of −1 g a vertical position. All values above 0.5 g were considered standing or moving. By summing up the absolute differences in acceleration values of the y-axis (for a 24 h period) total daily activity was calculated. 

The software settings were applied according to Stachowicz et al. [40]. The raw data were exported as CSV files to a computer using MSR software (version 6.6.02; MSR Electronics GmbH, Seuzach, Switzerland). To calculate the outcome variables of goats’ locomotion behavior, R (version 3.6.2; R Core Team, 2019, https://www.r-project.org; accessed on 1 January 2020) was used.

### 2.6. Statistical Analysis

The software R (version 3.6.2; R Core Team, 2019) was used for all statistical analyses. In total, eight linear mixed effects models and generalized linear mixed effects models were calculated by applying the functions lmer and glmer (package lme4 [42]). Fixed effects (explanatory variables) were based on hypotheses and prior descriptive analyses. They were determined in advance and are presented in Table 3. Random effects were used to correct for dependencies within farms and season and to correct for repeated observations of the same goat. The factor “INT (season + alpine pasture)” was created to consider the interactive effect of the two explanatory variables “season” and “alpine pasture”. It was not possible to include both fixed effects and their interaction in the model because the combination “spring + alpine pasture” did not occur. The three levels of the factor “INT” were thus “autumn + alpine pasture”, “autumn + no alpine pasture” and “spring + no alpine pasture”. The factor “INT (season + alpine pasture)” correlated strongly with the factor “pasture at time of data collection”.

The model assumptions were tested by visually inspecting model residuals for deviations from normality or homogeneity of variance. Due to deviations from model assumptions, the outcome variables activity (in 24 h) and lying bouts (in 24 h) were log transformed. Dummy variables with sum contrasts for all fixed effects were used. First, the full model comprising all fixed effects of interest was compared with the zero model (without any fixed effects). Further steps were only carried out if this comparison resulted in a *p*-value of < 0.05. *p*-values for the single fixed effects were obtained by comparing models reduced by this effect with the full model. To do so, a parametric bootstrap approach with 1000 simulations with the function PBmodcomp from the package pbkrtest [43] was used. Model estimates and confidence intervals were obtained with 1000 parametric bootstrap simulations (function bootMer of the lme4 package) [42]. The significance level was set to *p* = 0.05.

Before running the models, it was decided to replace the 25 claws with a severity of wall horn overgrowth score 0 with score 1 in order to obtain a binary distribution for further analyses. Overall, there were 2787 score 1 claws and 1957 score 2 claws. Animals that did not have their claws trimmed or had them only partly trimmed (animals that had been grazing on alpine pasture with claws with varying extent of wear) were erased (198 observations). Furthermore, it was decided to focus on the lesions “sole hemorrhage”, “horn separation” and “bleeding” as all other lesions (listed in File S3) were seen only occasionally. As a result of missing data for the time span since last trimming, 47 observations had to be removed from the analysis. Some locomotion raw data showed extreme deviations before and 2 weeks after trimming. This was due to changing management (farmers had the goats in indoor housing before trimming and on pasture two weeks later for the second logger recording or vice versa) and weather conditions (goats not being able to use the exercise yard because of strong snow fall) (18 whole farms). Other reasons for loss of data were pregnancy (eight goats), kidding (seven goats), stillbirth (two goats) and mastitis (one goat). Therefore, records of 18 individual animals and 18 whole farm records were excluded from the locomotion data. Lesions were recorded for each trimmed claw, and partly trimmed animals were recorded as trimmed animals. Thus, findings from 2445 trimmed claws of 309 goats in autumn and from 2062 trimmed claws of 260 goats in spring were analyzed.

## 3. Results

### 3.1. Claw Measurements

Measurements of sole length, sole width, toe length and heel height before and after trimming are shown in Table 4.

### 3.2. Wall Horn Overgrowth

The severity (score 0, 1 or 2), direction (axial wall, abaxial wall, both walls) and origin (axial wall, abaxial wall, both walls) of overgrowing wall horn for all examined claws (*n* = 2688) are shown in Table 5. In autumn, nearly all examined claws (98.2%) showed wall horn overgrowth, and moderate overgrowth predominated; in spring, all claws showed wall horn overgrowth, and severe overgrowth prevailed. The direction of overgrowing wall horn was mainly to the axial side, followed by both sides. Overgrown wall horn only to the abaxial side was seldom. The origin of overgrown wall horn was usually the abaxial wall. Overgrowing wall horn originating from both walls was recorded second most frequently, and wall horn overgrowth originating from the axial wall occurred very rarely.

**Figure 2:**
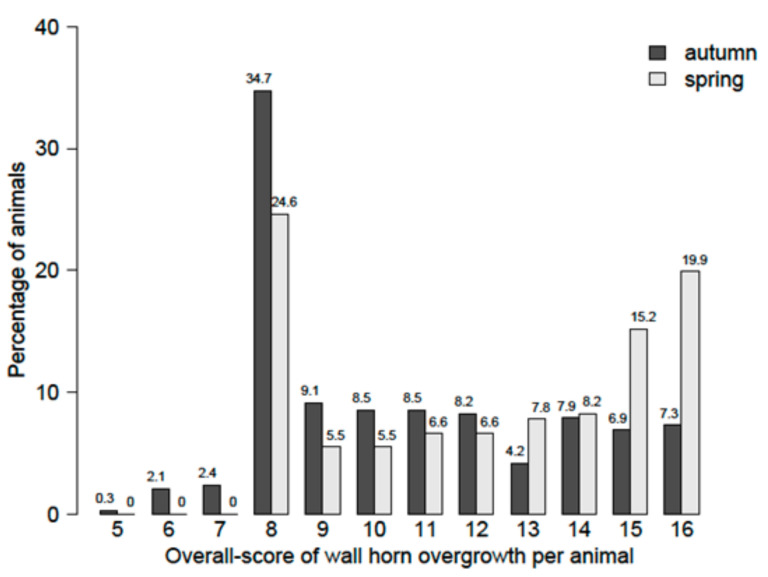
Distribution of the overall score of wall horn overgrowth per animal in autumn and spring.

The distribution of the overall score of wall horn overgrowth per animal (sum of all wall horn overgrowth scores of an animal) in autumn and spring is shown in Figure 2. In autumn, overall-score levels of wall horn overgrowth were lower than in spring.

**Figure 3:**
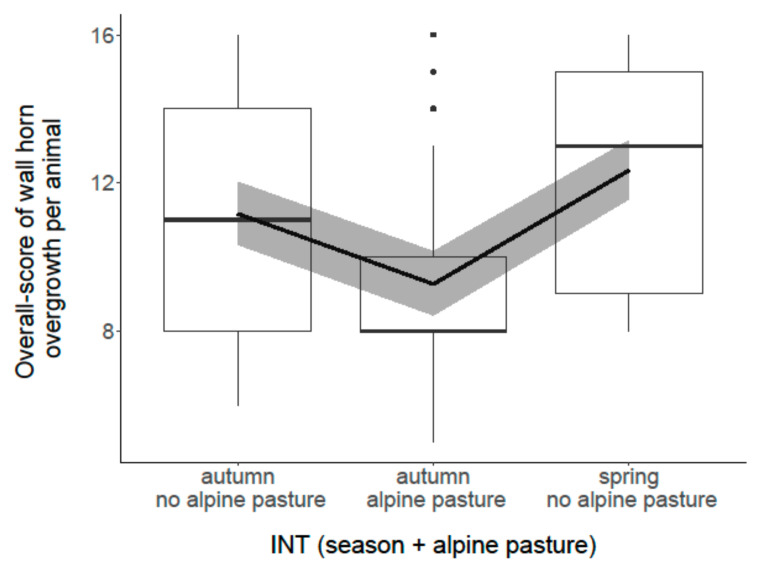
Association between “overall score of wall horn overgrowth per animal” and the factor “INT (season + alpine pasture)”. Box plot shows medians and interquartiles.. The dots represent outlier data points. The black line represents the model estimate, the gray colored area the 95% confidence interval.

**Figure 4:**
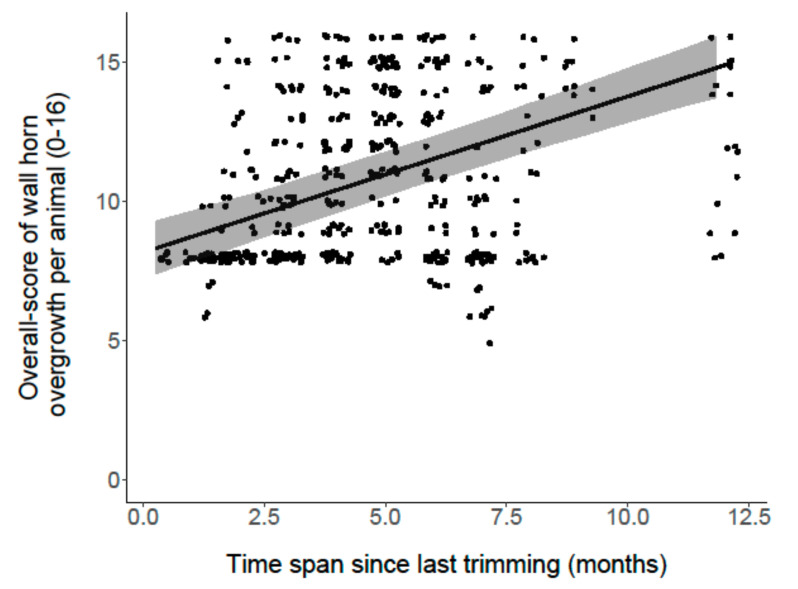
Association of “time span since last trimming” (in months) and “wall horn overgrowth per animal”. Dots show jittered raw data. The black line represents the model estimate, the gray colored area the 95% confidence interval.

The analysis of the effects of housing and management factors on wall horn overgrowth revealed that the overall score per animal was associated with the factor “INT (season + alpine pasture)” (*p* < 0.001; Figure 3). Goats that grazed on alpine pasture in summer showed less overgrowth compared with goats that did not graze on alpine pasture in summer and compared with all goats in spring. Furthermore, the model showed a correlation with “time span since last trimming” (*p* < 0.001; Figure 4). The more time had passed since the last trimming, the more wall horn overgrowth appeared. No association was found with “herd size” (*p* = 0.72) and “special skills training” (*p* = 0.61).

### 3.3. Slipper Foot

Slipper foot appeared on seven of the 28 farms. In autumn and spring combined, 13 goats (2.2%) showed at least one claw with slipper foot. In autumn, 1.0% (14 of 1340) and in spring 0.2% (2 of 1096) of all examined feet were diagnosed with slipper foot.

### 3.4. Claw Lesions

Data on claw lesions are shown in File S4 (Table S3), separately for autumn and spring recordings. The most common lesion was horn separation with 85.8% (265 of 309) and 87.3% (227 of 260) of the animals being affected in autumn and spring, respectively. The second most common lesion was sole hemorrhage, detected in 49.8% (154 of 309) of the goats in autumn and in 53.5% (139 of 260) in spring. A total of 389 bleeding claws due to injuries caused by trimming were detected. In autumn, 36.6% (112 of 309) of the goats and in spring 36.7% (95 of 260) of the goats had at least one such bleeding. Detailed information on horn separation, sole hemorrhage and bleedings can be found in File S4 (Table S4). Other claw lesions, such as horn fissures, ulcers, or foreign bodies, occurred only occasionally.

Only 10 goats on seven farms showed signs of inflammation of the interdigital space. Swabs (*n* = 11) were taken from these animals and tested for *D. nodosus*. All swabs were tested negative for virulent *D. nodosus*. On one farm, two swabs tested positive for benign *D. nodosus*. No lesions were found indicating infection with *Treponema* spp. Therefore, no swabs were taken to rule out infection with this pathogen.

#### 3.4.1. Sole Hemorrhages

**Figure 5:**
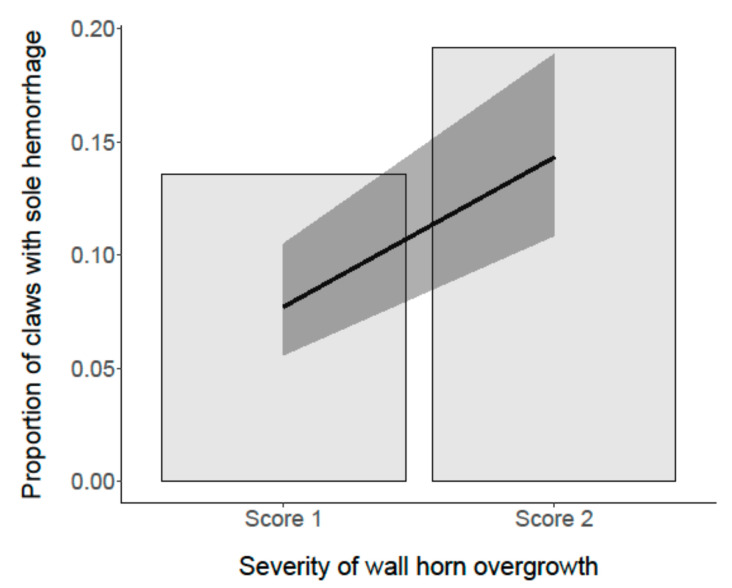
Association between the “severity score of wall horn overgrowth” and the proportion of claws with sole hemorrhage. The black line represents the model estimate, the gray colored area the 95% confidence interval.

**Figure 6:**
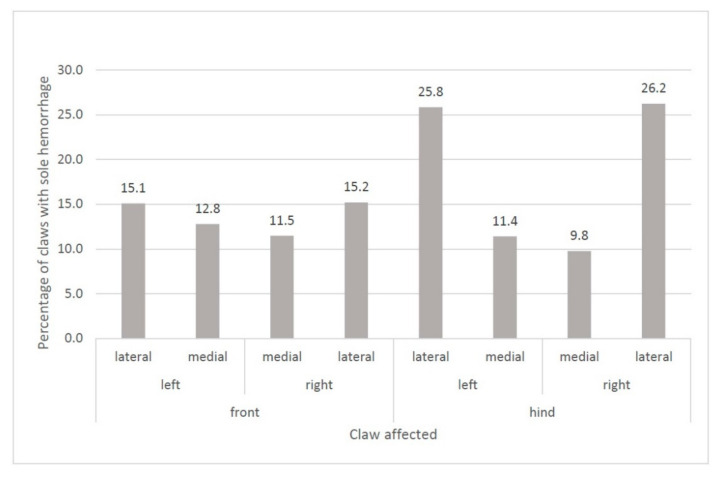
Localization of sole hemorrhages.

The “severity of wall horn overgrowth” was associated with the appearance of sole hemorrhages (*p* < 0.001; Figure 5). If claws showed severe overgrowth (score 2), the risk of developing sole hemorrhages was twice as high as for moderately overgrown claws (score 1). Furthermore, lateral hind claws were affected more frequently by sole hemorrhages than the medial hind claws and both lateral and medial front claws (Figure 6).

#### 3.4.2. Horn Separation

**Figure 7:**
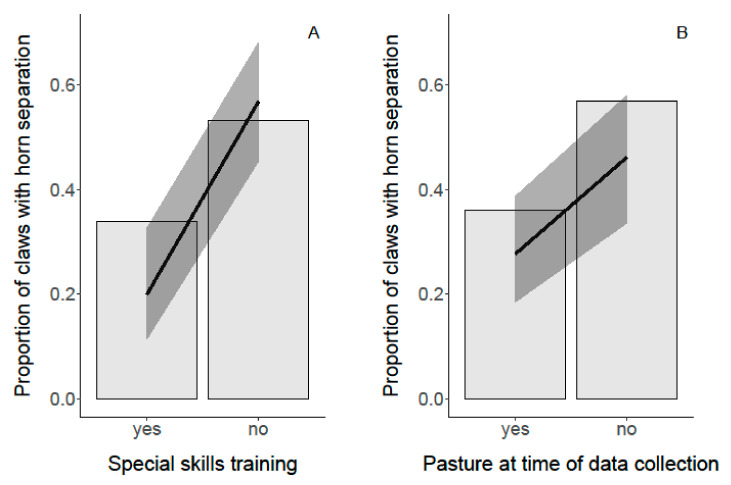
Association between “special skills training” (**A**) and “pasture at time of data collection” (**B**) and the proportion of claws with horn separation. The black lines represent the model estimates, the gray colored areas the 95% confidence intervals.

The factor “special skills training” was associated with the proportion of claws with horn separation (*p* < 0.001; Figure 7). If the trimmers had not participated in special skills training, the goats showed more claws with horn separation. In 80.8% of the claws with horn separation, claw trimming was done by farmers without special skills training. Conversely, trimmers with special skills training excised the lose horn more often (in 90% of the examined cases) and in accordance with claw trimming guidelines (*p* < 0.05). “Pasture at time of data collection” also was related with the appearance of horn separation (*p* < 0.001; Figure 7). Farms that had their goats graze on pasture at the time of data collection had fewer goats with horn separation. The “severity of wall horn overgrowth” was not associated with the proportion of claws with horn separation (*p* = 0.95).

#### 3.4.3. Bleeding

**Figure 8:**
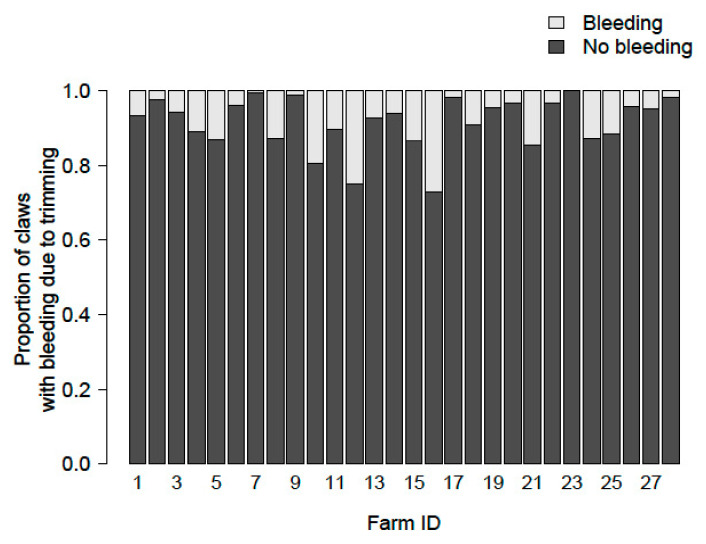
Distribution of claws with bleeding due to trimming on the 28 farms.

For bleeding caused by the trimmer during trimming, the comparison between the full model and the zero model did not show a difference (*p* = 0.19), indicating that neither the “severity of wall horn overgrowth” nor “special skills training” were associated with this type of lesion. However, the random effects implied a rather large variance among farms (Figure 8).

### 3.5. Locomotion

**Figure 9:**
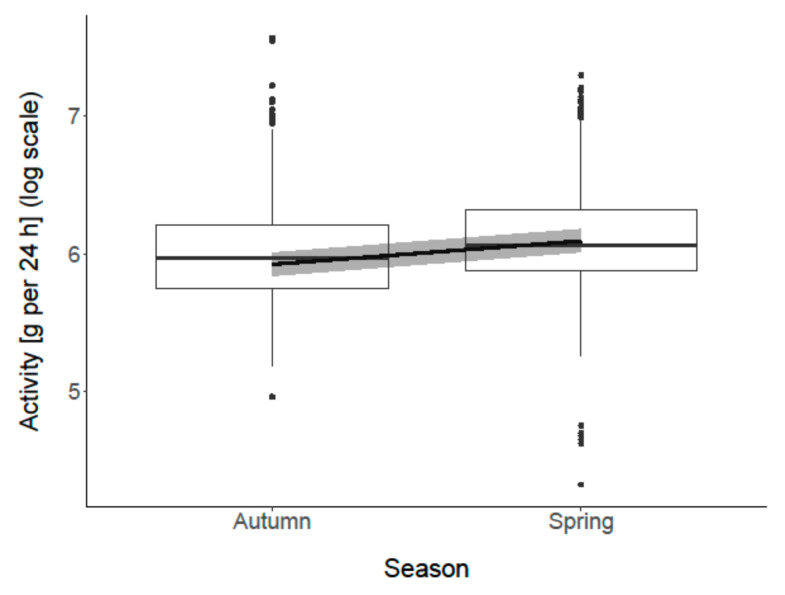
Association between “season” and goats’ activity (per 24 h). Activity (g = m/s^2^) is log transformed. Box plots show medians and interquartiles. The black line represents the model estimate, the gray colored area the 95% confidence interval.

“Season” was associated with the goats’ activity (recorded acceleration within 24 h, representing standing and moving) (*p* < 0.001; Figure 9). In spring, the goats were more active than in autumn. “Trimming (before/after)” was not associated with goats’ activity (*p* = 0.5).

**Figure 10:**
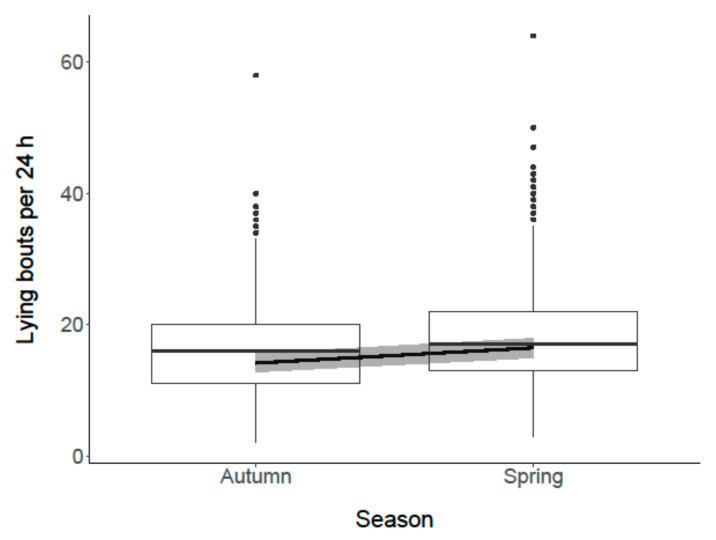
Association between “season” and the number of lying bouts (per 24 h) of the goats. Box plots show medians and interquartiles. The black line represents the model estimate, the gray colored area the 95% confidence interval.

Regarding total lying time, the comparison of the full model and the zero model did not show a difference (*p* = 0.2). Thus, “season” and “trimming (before/after)” were not associated with the total time goats spent lying in 24 h. However, the goats had more lying bouts in spring than in autumn (*p* < 0.001; Figure 10). “Trimming (before/after)” did not influence the number of lying bouts (*p* = 0.17).

## 4. Discussion

Overgrown wall horn can not only result in claw deformation and claw lesions, but can also cause abnormal gait and undue stress on joints, tendons and ligaments. In the long run, these effects can lead to lameness [18,19]. To the best of the author’s knowledge, this is the first study to collect detailed information on the severity, direction and origin of wall horn overgrowth on dairy goat claws. Of the examined claws, 98.2% and 100% were affected with wall horn overgrowth in autumn and in spring, respectively. This is in accordance with findings of Hill et al. [11], based on data from four dairy goat farms in England. In their study, 91.2% of the goats had at least one foot with wall horn overgrowth. They found differences between the farms, but on all farms the goats had wall horn overgrowth. Likewise, Anzuino et al. [11] reported that in 60.5% to 91.5% of the goats on 24 dairy goat farms in England, one or more feet were affected by wall horn overgrowth. Thus, wall horn overgrowth is a rather big issue on dairy goat farms and its consequences should be addressed more. 

With dairy cows, it has been shown that poor hoof conformation is a predisposition for hoof lesions and lameness [27,28]. Accordingly, several authors concluded that wall horn overgrowth is related to claw lesions, claw deformation and lameness in goats, too [12,18,19]. Furthermore, Grenho Ajuda et al. [44] investigated goat claws with thermography and showed that wall horn overgrowth and deformation can cause deep inflammation. Consequently, overgrown wall horn is likely to cause pain due to the inflammatory processes, at least temporarily. Grenho Ajuda et al. [44] also observed a reduction in claw temperature when deformed claws were trimmed and concluded that trimming reduces the severity of inflammation. This emphasizes the need for trimming to avoid poor hoof conformation.

In the present study, the intervals between routine trimmings varied widely (0.25–12 months, data not shown), and, as expected, the time span that had passed since the last trimming proved to have an effect on wall horn overgrowth. However, such overgrowth was found in almost all goats evaluated, indicating that the trimming regimens were not adapted to the housing conditions on the study farms. In more detail, goats kept indoors in winter on deep litter with access to an outdoor exercise yard had more wall horn overgrowth than goats grazing in valley areas in summer, and goats grazing on alpine pastures in summer had the least wall horn overgrowth. Thus, an indoor area with hard floor and/or an outdoor exercise yard does not ensure sufficient claw horn wear, and access to alpine pasture does not completely prevent wall horn overgrowth. Nevertheless, our results indicate that claw wear in goats on alpine pastures is enhanced due to prolonged contact between the claw and hard soil. Similar results were obtained by Ibrahim et al. [18] with sheep in extensive grazing systems. In conclusion, the results of the present study underline the need for adequate, regular and careful claw trimming in dairy goats, because wall horn overgrowth occurred on almost all examined claws.

The severity of wall horn overgrowth had an influence on the appearance of sole hemorrhages in the goats on the study farms. If claws were severely overgrown, the risk of developing sole hemorrhages was twice as high as that of claws with moderate overgrowth. Possibly, the non-physiological pressure concentration (in a certain claw area) increases with more pronounced wall horn overgrowth, resulting in damage of the corium followed by the emergence of hematomas. Similarly, for cows, van der Tol et al. [45] assumed that local pressure concentrations in animals with deformed claws walking on hard floor causes tissue overloading, leading to corium damage and eventually to hematomas and abnormal horn formation. Regarding the localization of sole hemorrhages on the feet of the herein studied goats, the percentage of claws with such lesions was highest for the lateral hind claws. Thus, a higher load is possibly exerted on the lateral compared with the medial hind claws in goats. With dairy cattle, van der Tol et al. [26] showed that, before trimming, 80% of the total hind limbs’ force was absorbed by the lateral claw, the remaining 20% by the medial claw. In conclusion, special attention should be put in avoiding severe wall horn overgrowth, especially on the hind claws.

Horn separations, sole hemorrhages and bleedings were the most frequent claw lesions recorded on the 28 study farms. Other types of lesions occurred only sporadically or not at all. Horn separation had the highest prevalence. The extent of the gap between wall and sole horn varied, as did the depth of the separation. Gelasakis et al. [46] described this type of claw lesion in sheep as “white line disease”, which also starts with a separation of the hoof wall from the laminar corium. The etiology of wall horn separation in sheep and goats is attributed to factors such as nutrient deficiencies, quality of ground and bedding, and genetic factors [38,46]. Hill et al. [11] argued that wall horn separation is not caused by a metabolic event and does not have a genetic basis, but is related to external factors. In the linear mixed effects model of the present study, the random effect of the individual animal for the occurrence of horn separation in a given claw was close to zero. This result indicates that horn separation in a single claw occurred independently from other claws of an individual goat and was thus likely not induced by metabolic causes.

In the present study, “special skills training” was correlated with the proportion of claws with horn separation. Goats trimmed by farmers without special skills training had a five times higher risk of showing horn separation than those trimmed by trained trimmers. In addition, farmers with “special skills training” excised the horn separation properly in 90% of the cases. With correct and careful excision, further horn separation is stopped, and the risk of an ascending infection or foreign body intrusion is reduced. Consequently, educating the farmers and providing special skills training is a crucial measure to improve claw health in dairy goats.

The third most common lesion was bleeding due to trimming. The statistical analysis did not support the assumption that trimmers with special skills training were more sensible to the topic and did the trimming more carefully in order to prevent bleeding. In addition, the suspicion that severely overgrown claws would be trimmed more vigorously in an attempt to remove as much overgrown wall horn as possible and to delay the next trimming could not be verified statistically. However, there were big differences between the 28 farms. Trimmers that had caused high numbers of bleeding in autumn tended to do so again in spring. Therefore, bleeding due to trimming seemed to be linked to personal trimming style. In the literature, emphasis is placed on avoiding bleeding when trimming healthy feet [18,34,35,47,48] to prevent pain and lesions such as abscesses, inflammation, and granulomatous lesions.

In sheep, infectious claw diseases are an important topic, and there are a few reports of foot rot infested goat claws all over the world [11,12,22,23,24]. Little is known yet about the presence of infectious claw diseases in Swiss goat herds. In the present study, only few goats with suspicious lesions were found. Of 11 swabs taken from inflamed interdigital spaces, all were tested negative with polymerase chain reaction for virulent *D. nodosus.* This finding is in accordance with the results of Ardüser et al. [49], who investigated the role of goats, cattle and South American camelids in the spread of foot rot in Switzerland. Their study proved goats did not play a role in the epidemiology of foot rot.

The goats’ locomotion behavior was recorded over a total of 12 days, before and after trimming in autumn and spring. It was expected that wall horn overgrowth would impair the goats’ locomotion to some extent and that they would therefore show more locomotion behavior after trimming. However, an effect of trimming on the goats’ activity and the total lying time could not be demonstrated. Possibly, the soft flooring (deep bedding indoors) mitigated the effects of non-physiological weight bearing and pressure concentration in claws with wall horn overgrowth, and therefore the goats’ locomotion behavior was not impaired. In the literature, there is no information on the influence of trimming on locomotion in goats. However, Ibrahim et al. [18] reported a significant increase in walking, feeding, drinking and ruminal activity in sheep after trimming.

Season was associated with the goats’ activity and the number of lying bouts per day. In spring, activity was higher and lying bouts were more frequent than in autumn. This difference could be due to their housing situation. Goat herds are structured very strictly by an established, quite stable linear hierarchy order [50]. In autumn, the goats on most farms had access to pasture with plenty of space. In spring, however, the animals were typically housed indoors and without access to pasture. This situation could have led to more agonistic interactions due to the reduced space allowance, associated with increased activity and more frequently disturbed lying bouts. In support of this interpretation, Barroso et al. [50] found a significant increase in agonistic behavior in goats while in the stable than while being shepherded. In the present study, data was collected in one spring and autumn respectively. To reveal whether spring/autumn characteristics of other years (e.g., other weather conditions, feed quality, heard health problems) would change the results concerning the effect of season on the goats’ activity a study over several years would be necessary.

## 5. Conclusions

Because wall horn overgrowth was observed in almost all examined claws, to a certain extent wall horn overgrowth in dairy goat claws is difficult to avoid under housing conditions common on Swiss dairy goat farms (indoor housing on deep litter during winter, pasture in the daytime during summer). The results of the present study show that wall horn overgrowth is associated with an increase in the proportion of claws with sole hemorrhages. Therefore, claw trimming should be performed regularly and at intervals that consider the housing conditions on a given farm and at different times of the year (i.e., access to pasture in summer, indoor housing in winter). Special skills training should be encouraged for claw trimmers because this helps to reduce horn separation. Wall horn overgrowth did not impair the goats’ locomotion behavior, as revealed by a comparison before and after trimming. However, housing conditions did not vary considerably between the 28 farms selected for this study. Therefore, further studies should be conducted to investigate the effects of claw conformation, claw health and wall horn overgrowth on locomotion behavior in dairy goats. Regarding animal welfare, further studies should also consider how lameness is influenced by wall horn overgrowth, claw lesions and housing conditions.

## Figures and Tables

**Table 1:** Classification of wall horn overgrowth (NA = not applicable).

Assessment	0	1	2	3
Severity of wall horn overgrowth (scores 0–2, per claw)	no overgrowth	moderate; <50% of sole surface covered by overgrown wall horn	severe; >50% of sole surface covered by overgrown wall horn	NA
Overall score of wall horn overgrowth	0–16; summed-up score per animal			
Direction of overgrowth	NA	to the axial side	to the abaxial side	to the axial and abaxial side
Origin of overgrowth	NA	abaxial wall horn	axial wall horn	axial and abaxial wall horn

**Table 2:** List of the lesions documented on the claw card, their definition supplemented with pictures and references.

Lesion	Definition	Picture	Literature
Bleeding (due to trimming)	The lamina is injured, with blood extravasating	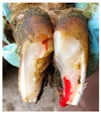	
Chronic laminitis	Horizontal grooves in the wall horn, as a result of an acute laminitis with vascular injury of the corium (appears weeks up to months later)		[35,36]
Foreign body	Any small object (e.g., stone, nail) that is lodged into the claw	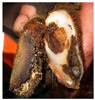	[11]
Granulomatous lesion	Raspberry-like proliferation of the lamina	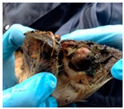	[11,35]
Heel horn erosion	Erosion of the heel, in severe cases typically V-shaped, possibly extending to the corium		[37]
Horn fissure	Horizontal crack in the claw wall; vertical (longitudinal) crack in the outer or dorsal claw wall	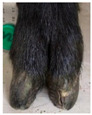	[37]
Horn separation	The wall and sole of the foot have grown apart. The space between the wall and sole varies, as does the depth of separation	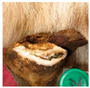	[11,38]
Interdigital phlegmon	Symmetric painful swelling of the foot commonly accompanied by foul smell, with sudden onset of lameness	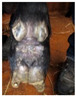	[37]
Sole hemorrhage	Diffused light red to yellowish discoloration; clearly distinguishable from normal colored horn	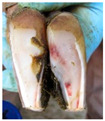	[37]
Sole/toe abscess	Abscess due to lesion and bacterial infection of the lamina	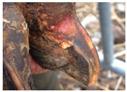	[35]
Sole/toe ulcer	Ulceration of the sole area specified according to localization such as sole ulcer, toe ulcer	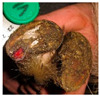	[37]
Interdigital space inflammation score 1	Mild dermatitis, without necrosis	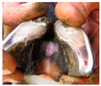	
Interdigital space inflammation score 2	Severe dermatitis, with necrosis	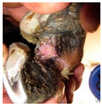	

**Table 3:** Fixed effects (explanatory variables).

Outcome Variable	Description	Fixed Effects	Random Effects
Overall score of wall horn overgrowth per animal	One score per goat (0–16)	INT (season + alpine pasture), herd size, time span since last trimming, special skills training (yes/no)	Observation < goat < farm
Sole hemorrhages	One finding per claw	Severity of wall horn overgrowth	Observation < season < goat < farm
Horn separation		Severity of wall horn overgrowth, special skills training (yes/no), pasture at time of data collection	
Horn separation (excised)		Special skills training (yes/no)	
Bleeding		Severity of wall horn overgrowth, special skills training (yes/no)	
Total lying time in 24 h	4 × 72 h of observation per goat	Trimming (before/after), season (spring/autumn)	Observation < trimming (before/after) < season < goat < farm
Number of lying bouts in 24 h			
Activity in 24 h			

**Table 4:** Measurements (mean and standard deviation) and differences (in centimeters) of the medial and lateral claw of the front left and hind right foot from 604 goats before and 617 goats after trimming.

Foot	Assessment		Before Trimming		After Trimming		Difference
Front Left	Sole length (cm)	Lateral Medial	4.8 4.9	(±1.57) (±1.61)	4.3 4.4	(±1.27) (±1.30)	0.5 0.5
	Sole width (cm)	Lateral Medial	2.2 2.4	(±0.74) (±0.80)	2.1 2.2	(±0.63) (±0.65)	0.1 0.2
	Toe length (cm)	Lateral Medial	4.0 4.0	(±1.32) (±1.32)	3.5 3.4	(±1.03) (±1.00)	0.5 0.6
	Heel height (cm)	Lateral Medial	3.8 3.7	(±1.24) (±1.18)	3.5 3.3	(±1.02) (±0.97)	0.3 0.4
Hind right	Sole length (cm)	Lateral Medial	5.0 4.8	(±1.66) (±1.56)	4.3 4.2	(±1.26) (±1.23)	0.7 0.6
	Sole width (cm)	Lateral Medial	2.1 2.1	(±0.70) (±0.73)	1.9 1.9	(±0.58) (±0.59)	0.2 0.2
	Toe length (cm)	Lateral Medial	4.0 4.0	(±1.63) (±1.36)	3.2 3.3	(±0.79) (±0.98)	0.8 0.7
	Heel height (cm)	Lateral Medial	3.3 3.2	(±1.00) (±1.04)	3.0 3.0	(±0.85) (±0.85)	0.3 0.2

**Table 5:** Severity (score 0, 1 or 2), direction (no overgrowth, axial, abaxial or both walls) and origin (no overgrowth, axial, abaxial or both walls) of wall horn overgrowth recorded in autumn and spring (NA = information not available).

Wall Horn Overgrowth		Autumn		Spring	
		Number of Claws	Percentage of Claws	Number of Claws	Percentage of Claws
Severity	Score 0 Score 1 Score2 NA	25 1777 864 22	0.93% 66.11% 32.14% 0.82%	0 985 1093 610	0% 36.64% 40.66% 22.70%
Direction	No overgrowth Axial wall Abaxial wall Both walls NA	25 2166 9 466 22	0.93% 80.58% 0.33% 17.34% 0.82%	0 1514 10 554 610	0% 56.32% 0.37% 20.61% 22.70%
Origin	No overgrowth Axial wall Abaxial wall Both walls NA	25 18 2005 618 22	0.93% 0.67% 74.59% 22.99% 0.82%	0 4 1426 648 610	0% 0.15% 53.05% 24.11% 22.69%

## Data Availability

The datasets generated and/or analyzed during the current study are not publicly available due to the individual privacy of goat owners, but are available from the corresponding author on reasonable request.

## Supplementary Materials

The following are available online at https://www.mdpi.com/article/10.3390/ani11071873/s1, Additional File S1: Table S1 Housing, management and trimming quality assessment questionnaire. File S2: Table S2 Questionnaire for 28 farms. Abbreviations: (+) = yes, (-) = no, NA = no information available, FS = foot shears, PS = pruning shears, HK = hoof knife, PK = pocket knife, AG = angle grinder. File S3: Claw card (translated to English); File S4: Table S3: Claw lesions as documented on the claw cards in autumn and spring. Claws affected and animals affected. Autumn: 2445 trimmed claws and 309 animals; spring: 2062 trimmed claws and 260 animals, Table S4 Animals affected with horn separation, sole hemorrhage and with bleeding due to trimming in autumn and spring, with number of claws affected per animal, Table S5: Animals affected with sole hemorrhage in autumn and spring, with number of claws affected per animal., Table S6: Animals affected with bleeding due to trimming in autumn and spring, with number of claws affected per animal.

## References

[1] Miller B.A., Lu C.D. (2019). Current status of global dairy goat production: An overview. Asian-Australas. J. Anim. Sci..

[2] Park Y.W. (1994). Hypo-allergenic and therapeutic significance of goat milk. Small Rumin. Res..

[3] International Diabetes Federation (2017). The World Dairy Situation 2017. Bulletin of International Dairy Federation 489/2017.

[4] Food and Agriculture Organization of the United Nations Statistical Databases. http://www.fao.org/statistics/en/.

[5] McGonegal M. (2017). Dairy goats in Ontario: A growing industry. Canadian Agriculture at a Glance.

[6] Herren U. (2019). Schweizerischer Ziegenzuchtverband Jahresbericht 2018 [Swiss Goat Breeders Association Annual Report 2018].

[7] TSM, Schweizer Milchproduzenten, Switzerland Cheese Marketing, Branchenorganisation Milch, Agristat (2019). Milchstatistik der Schweiz 2018 [Dairy statistics Switzerland 2018].

[8] Escareño L., Salinas-Gonzalez H., Wurzinger M., Iñiguez L., Sölkner J., Meza-Herrera C. (2012). Dairy goat production systems: Status quo, perspectives and challenges. Trop. Anim. Health Prod..

[9] Zobel G., Neave H.W., Webster J. (2018). Understanding natural behavior to improve dairy goat (*Capra hircus*) management systems. Transl. Anim. Sci..

[10] Popescu A. (2013). Study refarding the trends in the World and European goat milk production. Lucr. Stiintifice Ser. Zootehine.

[11] Hill N.P., Murphy P.E., Nelson A.J., Mouttotou N., Green L.E., Morgan K.L. (1997). Lameness and foot lesions in adult British dairy goats. Vet. Rec..

[12] Christodoulopoulos G. (2009). Foot lameness in dairy goats. Res. Vet. Sci..

[13] Anzuino K., Bell N.J., Bazeley K.J., Nicol C.J. (2010). Assessment of welfare on 24 commercial UK dairy goat farms based on direct observations. Vet. Rec..

[14] Kofler J. (2016). Klauenpflege bei Schaf und Ziege [Claw trimming on sheep and goat]. Klauentrierpraxis.

[15] Gautam M., Stevenson M.A., Lopez-Villalobos N., McLean V. (2017). Risk factors for culling, sales and deaths in New Zealand dairy goat herds, 2000–2009. Front. Vet. Sci..

[16] Molares F.A., Genis J., Mena Y. (2019). Current status, challenges and the way forward for dairy goat production in Europe. Asian-Australs. J. Anim. Sci..

[17] Lu C.D., Miller B.A. (2019). Current status, challenges and prospects for dairy goat production in the Americas. Asian-Australs. J. Anim. Sci..

[18] Ibrahim A., Mahmoud U.T., Khalil N.S.A., Hussein H.A., Ali M.M. (2018). A pilot study on surgical trimming impact on severely overgrown claws in sheep: Behavioral, physiological, and ruminal function aspects. J. Vet. Behav..

[19] Smith M.C., Sherman D.M. (2009). Goat Medicine.

[20] Groenevelt M., Anzuino K., Langton D.A., Grogono-Thomas R. (2015). Association of treponeme species with atypical foot lesions in goats. Vet. Rec..

[21] Crosby-Durrani H.E., Clegg S.R., Singer E., Angell J.W., Evans N.J., Carter S.D., Blundell R.J., Duncan J.S. (2016). Severe foot lesions in dairy goats associated with digital dermatitis treponemes. J. Comp. Pathol..

[22] Aguiar G.M.N., Simões S.V.D., Silva T.R., Assis A.C.O., Medeiros J.M.A., Garino F., Riet-Correa F. (2011). Foot rot and other foot diseases of goat and sheep in the semiarid region of northeastern Brazil. Pesqui Vet. Brasil..

[23] Laven R. (2012). Use of parenteral long-acting and topical oxytetracycline, without hoof trimming, for treatment of footrot in goats. N. Z. Vet. J..

[24] Bennett G., van Loenen A., Zhou H., Sedcole R., Hickford J. (2009). The detection of *Dichelobacter nodosus* and *Fusobacterium necrophorum* from footrot lesions in New Zealand goats. Anaerobe.

[25] Mohamadnia A., Khaghani A. (2013). Evaluation of hooves’ morphometric parameters in different hoof trimming times in dairy cows. Vet. Res. Forum..

[26] van der Tol P.P.J., van der Beek S.S., Metz J.H.M., Noordhuizen-Stassen E.N., Back W., Braam C.R., Weijs W.A. (2004). The effect of preventive trimming on weight bearing and force balance on the claws of dairy cattle. J. Dairy Sci..

[27] Kaler J., Medley G.F., Grogono-Thomas R., Wellington E.M.H., Calvo-Bado L.A., Wassink G.J., King E.M., Moore L.J., Russell C., Green L.E. (2010). Factors associated with changes of state of foot conformation and lameness in a flock of sheep. Prev. Vet. Med..

[28] Boettcher P.J., Jairath L.K., Koots K.R., Dekkers J.C.M. (1997). Effects of interactions between type and milk production on survival traits of Canadian Holsteins. J. Dairy Sci..

[29] Koluman N., Göncü S. (2016). Measurements of healthy hooves, their interrelation and correlation with body mass in some improved goat breeds. Int. J. Agric. Environ. Biotechnol..

[30] Grenho Gonçalves Ajuda I., Battini M., Stilwell G.T. (2019). The role of claw deformation and claw size on goat lameness. Vet. Anim. Sci..

[31] Quirk T.J. (2016). Excel 2016 for Social Science Statistics: A Guide to Solving Practical Problems.

[32] Bhardwaj V., Dhungyel O.P., de Silva K., Dhand N.K., Whittngton R.J. (2018). An objective method for assessment of foot conformation in sheep. Small Rumin. Res..

[33] Lottner S. (2006). Felduntersuchung zur Bekämpfung der Moderhinke bei Schafen Mittels Vakzinen und Genetischer Marker [Field Study for Fighting Foot Rot in Sheep by Means of Vaccines and Genetic Markers]. Ph.D. Thesis.

[34] Winter A.C. (2011). Treatment and control of hoof disorders in sheep and goats. Vet. Clin. N. Am. Food Anim. Pract..

[35] Strobel H. (2014). Klauenpflege Schaf und Ziege [Claw Trimming Sheep and Goat].

[36] Katsoulos P.-D., Giadinis N.D., Chaintoutis S.C., Dovas C.I., Kiossis E., Tsousis G., Psychas V., Vlemmas I., Papadopoulos T., Papadopoulos O. (2016). Epidemiological characteristics and clinicopathological features of bluetongue in sheep and cattle, during the 2014 BTV serotype 4 incursion in Greece. Trop. Anim. Health Prod..

[37] ICAR Working Group on Functional Traits and International Claw Health Experts (2015). (Ed.) ICAR Claw Health Atlas.

[38] Winter A.C. (2008). Lameness in sheep. Small Rumin. Res..

[39] Stäuble A., Steiner A., Frey J., Kuhnert P. (2014). Simultaneous detection and discrimination of virulent and benign *Dichelobacter nodosus* in sheep of flocks affected by foot rot and in clinically healthy flocks by competitive real-time PCR. J. Clin. Microbiol..

[40] Stachowicz J., Gygax L., Hillmann E., Wechsler B., Keil N.M. (2018). Dairy goats use outdoor runs of high quality more regardless of the quality of indoor housing. Appl. Anim. Behav. Sci..

[41] Egli P. (2018). Aktivitätsverhalten von Milch- und Zwergziegen [Activity Behaviour of Dairy and Dwarf Goats]. Master’s Thesis.

[42] Bates D., Mächler M., Bolker B., Walker S. (2015). Fitting linear mixed-effects models using lme4. J. Stat. Softw..

[43] Halekoh U., Højsgaard S. (2014). A Kenward–Roger approximation and parametric bootstrap methods for tests in linear mixed models—the R package pbkrtest. J. Stat. Softw..

[44] Grenho Ajuda I., Vieira A., Stilwell G. Are there differences in dairy goats claws’ temperature, before and after trimming? In Proceedings of the IEEE International Symposium on Medical Measurements and Applications, Lisbon, Portugal, 11–12 June 2014.

[45] Van der Tol P.P.J., Metz J.H.M., Noordhuizen-Stassen E.N., Back W., Braam C.R., Weijs W.A. (2003). The vertical ground reaction force and the pressure distribution on the claws of dairy cow while walking on flat substrate. J. Dairy Sci..

[46] Gelasakis A.I., Kalogianni A.I., Bossis I. (2019). Aetiology, risk factors, diagnosis and control of foot-related lameness in dairy sheep. Animals.

[47] Winter A. (1997). Treatment of toe granuloma in sheep. In Practice..

[48] Hodgkinson O. (2010). The importance of feet examination in sheep health management. Small Rumin. Res..

[49] Ardüser F., Moore-Jones G., Gobeli Brawand S., Dürr S., Steiner A., Ryser-Degiorgis M.-P., Zanolari P. (2020). Dichelobacter nodosus in sheep, cattle, goats and South American camelids in Switzerland—assessing prevalence in potential hosts in order to design targeted disease control measures. Prev. Vet. Med..

[50] Barroso F.G., Alados C.L., Boza J. (2000). Social hierachy in the domestic goat: Effect on food habits and production. Appl. Anim. Behav. Sci..

