# Supplementary material for: Influence of Housing and Management on Claw Health in Swiss Dairy Goats
Source: Animals (Basel). 2021 Jun 23;11(7):1873. doi: 10.3390/ani11071873 (PMC8300172; doi:10.3390/ani11071873)
Supplement: Supplementary file 1 [file animals-11-01873-s001.zip › Additional Files/Additional File S1.pdf]

File S1

Table S1. Housing, management and quality assessment questionnaire.

**Housing**	
	Straw yard yes/no
	Additional area with hard floor yes/no
	Access to outdoor exercise yard yes/no
	Access to pasture in summer yes/no<br>Access to pasture at time of data collection (minimum of four weeks prior to data collection) yes/no
	Grazing on alpine pasture yes/no
Management	
	Frequency of routine claw trimming
	Age of goats when first trimming is conducted (in months)
	Time span since last trimming (how long ago was the last trimming in months)
	Who conducts the trimming (farmer him/herself, employee or an external, trained staff)
	Special skills training (has the trimmer ever attended a special training for trimming small ruminants' claws)
	Usage of footbaths and in what composition
Quality of trimming	
	Cutting back wall horn ok/excessive
	Bleeding lesions due to claw trimming
	Equipment used for trimming
	Disinfection of trimming equipment yes/no
	Disinfection of lesions yes/no
