# Supplementary material for: Influence of Housing and Management on Claw Health in Swiss Dairy Goats
Source: Animals (Basel). 2021 Jun 23;11(7):1873. doi: 10.3390/ani11071873 (PMC8300172; doi:10.3390/ani11071873)
Supplement: Supplementary file 1 [file animals-11-01873-s001.zip › Additional Files/Additional File S2.pdf]

Farm ID	*Herd size*	*Breed*	*Horn status*	*Straw yard*	*Additional area with hard floor*	*Excercise yard*	*Pasture*	*Grazing in alpine regions*	*Frequency of routine trimming/ year*	*Age of first trimming/ months*	*Who conducts the trimming (first /second trimming)*	*Special skills training (first /second trimming)*	*Application of footbaths*	*Equipment used (first/second trimming)*	*Disinfecting of bleeding lesions (first/second trimming)*
**1**	50	Mixed	Mixed	+	+	+	+	-	2-3	5	Farmer	-	-	FS	-
**2**	85	Mixed	Mixed	+	+	+	+	-	5-6	5-6	Employee	-	-	PS, PK	-
**3**	30	Chamois Colored	Hornless	+	+	+	+	-	3-4	5-6	Employee	+	-	FS	-
**4**	80	Mixed	Mixed	+	+	+	+	-	2	12	Farmer	-	-	PS	+/-
**5**	36	Mixed	Hornless	+	+	+	+	-	NA	4.5-6	Farmer	+	-	FS	-
**6**	60	Chamois Colored	Hornless	-	+	+	+	+	4-5	5	Farmer	-	-	PK	+/-
**7**	100	Saanen	Mixed	+	+	+	+	-	3	6	Farmer/Employee	+/-	-	PS	-
**8**	30	Mixed	Hornless	-	+	-		-	5-6	6	Farmer	-	-	PS, HK	-
**9**	30	Mixed	Mixed	+	+	+	+	-	5-6	3	Farmer	-	-	FS	-
**10**	70	Mixed	Mixed	+	+	+	+	+	2	6	Farmer	-	-	FS, PK/ FS, HK	-
**11**	20	Chamois Colored	Hornless	+	+	+	+	+	4-5	6	Farmer	+	-	FS, PK	-
**12**	24	Appenzeller	Hornless	+	+	-	+	+	4	6-9	Farmer	-	-	FS	-
**13**	25	Appenzeller	Hornless	+	+	+	+	+	NA	6	Farmer	-	-	PS	-
**14**	80	Toggenburger	Mixed	+	+	+	+	+	2	6	Farmer	-	-	HK	-
**15**	85	Mixed	Mixed	+	+	+	+	+	2	6	Farmer	-	-	FS, HK/ FS	+/-
**16**	75	Mixed	Hornless	-	+	+	+	-	3	5-6	Farmer	-	-	FS, AG/ FS, HK	-/+
**17**	280	Mixed	Mixed	+	-	+	-	-	3	5-6	Farmer	-	-	FS	+/-

**18**	60	Mixed	Hornless	+	+	+	+	-	NA	12	Farmer/Employee	+/-	-	PS, HK	+/-
**19**	20	Mixed	Horned	+	+	+	+	+	3-4	9-12	Farmer	-	-	HK/ FS, HK	-
**20**	64	Mixed	Hornless	+	+	+	+	-	2	20	Farmer	+	-	PS, HK/ PS, HK, FS	-
**21**	20	Mixed	Hornless	+	+	+	+	-	2	20	Farmer	-	-	PS, HK, FS/ GS	-/+
**22**	49	Mixed	Horned	+	+	+	+	+	1	12	Farmer	-	-	FS, HK	
**23**	50	Mixed	Horned	+	+	+	+	+	2-3	8-9	Farmer	+	-	FS, HK	(spray at hand)
**24**	100	Mixed	Mixed	+	+	+	+	-	2-3	NA	Hired staff/NA	-	-	FS, TK/ NA	+/NA
**25**	30	Mixed	Hornless	+	+	+	+	-	2	6-12	Hired staff/NA	+	-	FS, AG/ NA	+/NA
**26**	60	Saanen	Mixed	+	+	+	+	-	6	2-2.5	Farmer	-	-	FS, AG/ FS, PK	-
**27**	70	Mixed	Mixed	+	+	+	+	-	2-3	6-7	Farmer	-	-	FS	-
**28**	50	Mixed	Mixed	+	+	+	+	+	2	NA	Farmer	-	-	PS	(spray at hand)
**Mean**	61.9								3.1	7.8					

Table S2. Questionnaire for 28 farms. Abbreviations: (+) = yes, (-) = no, NA = no information available, FS= foot shears, PS= pruning shears, HK= hoof knife, PK= pocket knife, AG= angle grinder.
