# Supplementary material for: Influence of Housing and Management on Claw Health in Swiss Dairy Goats
Source: Animals (Basel). 2021 Jun 23;11(7):1873. doi: 10.3390/ani11071873 (PMC8300172; doi:10.3390/ani11071873)
Supplement: Supplementary file 1 [file animals-11-01873-s001.zip › Additional Files/Additional File S3.pdf]

Farm ID: ______________
Ear tag: ______________
Breed: ______________
Date: ______________

lat Front left med

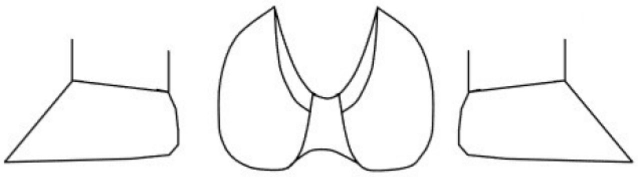

interdigital space
0 = unobtrusiv
1 = mild dermatitis without necrosis
2 = severe dermatitis accompanied with necrosis

prior	post		post	prior
		no pathological changes		
		bleeding due to trimming		
		chronic laminitis		
		foreign body		
		granulomatous lesion		
		heel horn erosion		
		horn fissure		
		horn separation		
		interdigital phlegmon		
		sole hemorrhage		
		sole/toe abscess		
		sole/toe ulcer		

med Front right lat

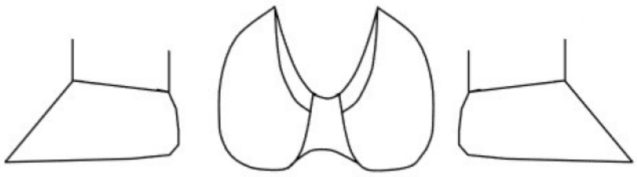

interdigital space
0 = unobtrusiv
1 = mild dermatitis without necrosis
2 = severe dermatitis accompanied with necrosis

prior	post		post	prior
		no pathological changes		
		bleeding due to trimming		
		chronic laminitis		
		foreign body		
		granulomatous lesion		
		heel horn erosion		
		horn fissure		
		horn separation		
		interdigital phlegmon		
		sole hemorrhage		
		sole/toe abscess		
		sole/toe ulcer		

lat Hind left med

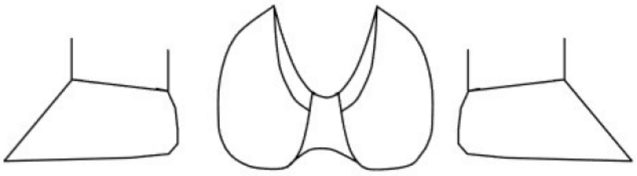

interdigital space
0 = unobtrusiv
1 = mild dermatitis without necrosis
2 = severe dermatitis accompanied with necrosis

prior	post		post	prior
		no pathological changes		
		bleeding due to trimming		
		chronic laminitis		
		foreign body		
		granulomatous lesion		
		heel horn erosion		
		horn fissure		
		horn separation		
		interdigital phlegmon		
		sole hemorrhage		
		sole/toe abscess		
		sole/toe ulcer		

med Hind right lat

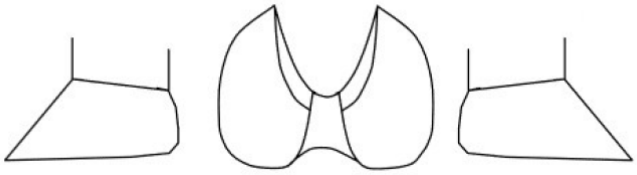

interdigital space
0 = unobtrusiv
1 = mild dermatitis without necrosis
2 = severe dermatitis accompanied with necrosis

prior	post		post	prior
		no pathological changes		
		bleeding due to trimming		
		chronic laminitis		
		foreign body		
		granulomatous lesion		
		heel horn erosion		
		horn fissure		
		horn separation		
		interdigital phlegmon		
		sole hemorrhage		
		sole/toe abscess		
		sole/toe ulcer		
