# Supplementary material for: Influence of Housing and Management on Claw Health in Swiss Dairy Goats
Source: Animals (Basel). 2021 Jun 23;11(7):1873. doi: 10.3390/ani11071873 (PMC8300172; doi:10.3390/ani11071873)
Supplement: Supplementary file 1 [file animals-11-01873-s001.zip › Additional Files/Additional File S4.pdf]

Additional File S4

Table S3 Claw lesions as documented on the claw cards in autumn and spring. Claws affected and animals affected. Autumn 2445 trimmed claws and 309 animals; Spring 2062 trimmed claws and 260 animals.

Lesion	*Autumn*				*Spring*			
	Claws		Goats		Claws		Goats	
Horn separation	1205	(49.5 %)	265	(85.8 %)	959	(46.5 %)	227	(87.3 %)
Sole Hemorrhage	344	(14.1 %)	154	(49.8 %)	376	(18.2 %)	139	(54.5 %)
Bleeding	228	(09.3 %)	112	(36.6 %)	161	(07.8 %)	95	(36.7 %)
Foreign body	8	(00.3 %)	8	(02.6 %)	9	(00.4 %)	9	(03.5 %)
Granulomatous lesion	3	(00.1 %)	2	(00.7 %)	0	(00.0 %)	0	(00.0 %)
Horn Fissure	2	(00.1 %)	2	(00.1 %)	0	(00.0 %)	0	(00.0 %)
Interdigital phlegmon	2	(00.1 %)	1	(00.3 %)	0	(00.0 %)	0	(00.0 %)
Toe ulcer	0	(00.0 %)	0	(00.0 %)	1	(00.1 %)	1	(00.4 %)

Table S4 Animals affected with horn separation in autumn and spring, with number of claws affected per animal.

**Number of claws per goat with horn separation**	**Goats affected in autumn**	**Goats affected in spring**
**0**	43	34
**1**	28	23
**2**	43	29
**3**	38	34
**4**	31	40
**5**	26	36
**6**	19	29
**7**	27	19
**8**	53	16

Table S5 Animals affected with sole hemorrhage in autumn and spring, with number of claws affected per animal.

Number of claws per goat with sole hemorrhage	Goats affected in autumn	Goats affected in spring
**0**	150	117
**1**	90	57
**2**	34	39
**3**	14	20
**4**	10	15
**5**	2	7
**6**	4	1

Table S6 Animals affected with bleeding due to trimming in autumn and spring, with number of claws affected per animal.

Number of claws per goat with bleeding	Goats affected in autumn	Goats affected in spring
**0**	196	165
**1**	52	60
**2**	26	17
**3**	21	8
**4**	7	7
**5**	4	3
**6**	6	0
